# Exposure–lag response of smoking prevalence on lung cancer incidence using a distributed lag non-linear model

**DOI:** 10.1038/s41598-021-91644-y

**Published:** 2021-07-14

**Authors:** Daniel Robert Smith, Alireza Behzadnia, Rabbiaatul Addawiyah Imawana, Muzammil Nahaboo Solim, Michaela Louise Goodson

**Affiliations:** 1 Newcastle University Medicine Malaysia, No. 1, Jalan Sarjana 1, Kota Ilmu, EduCity@Iskandar, 79200 Iskandar Puteri, Johor Malaysia; 2 Histopathology Department, Leeds Teaching Hospital, NHS Trust, Beckett Street, Leeds, LS9 7TF West Yorkshire UK; 3 The James Cook University Hospital, Marton Road, Middlesbrough, TS4 3BW UK

**Keywords:** Oncology, Risk factors

## Abstract

The prevalence of smokers is a major driver of lung cancer incidence in a population, though the “exposure–lag” effects are ill-defined. Here we present a multi-country ecological modelling study using a 30-year smoking prevalence history to quantify the exposure–lag response. To model the temporal dependency between smoking prevalence and lung cancer incidence, we used a distributed lag non-linear model (DLNM), controlling for gender, age group, country, outcome year, and population at risk, and presented the effects as the incidence rate ratio (IRR) and cumulative incidence rate ratio (IRR_cum_). The exposure–response varied by lag period, whilst the lag–response varied according to the magnitude and direction of changes in smoking prevalence in the population. For the cumulative lag–response, increments above and below the reference level was associated with an increased and decreased IRR_cum_ respectively, with the magnitude of the effect varying across the lag period. Though caution should be exercised in interpretation of the IRR and IRR_cum_ estimates reported herein, we hope our work constitutes a preliminary step towards providing policy makers with meaningful indicators to inform national screening programme developments. To that end, we have implemented our statistical model a shiny app and provide an example of its use.

## Introduction

Globally, lung cancer accounts for 11.6% and 18.4% of all cancer cases and deaths respectively^1^. In 2018, lung cancer accounted for 1.79 million deaths globally, with 2.09 million new cases diagnosed. Tobacco use is the primary cause of most lung cancers^2,3^, and although other methods of tobacco consumption have emerged, smoking remains by far the most common^4^.

The World Health Organization (WHO) estimates tobacco related mortality will increase from 100 million in the twentieth century to one billion in twenty-first century if current trends in smoking continue^3^. To circumvent this epidemic, the WHO Framework Convention on Tobacco Control (FCTC), the first ever global health treaty, was initiated in 2003, with the overarching goal of implementing effective policies to reduce tobacco consumption^5^. In 2013, WHO set the target of a 30% smoking prevalence reduction by 2025 in all 178 countries that signed the FCTC. Projections based on limited publicly available data have shown that less than half of these countries are likely to meet this target^2,6^. Between 2009 and 2017, smoking prevalence declined by 7.7% (male) and 15.2% (female) globally^7^. This decline has been the trend for the majority of high-income countries^8^, though some nations (e.g. Males in Albania; Females in Portugal) have seen substantial increases during this period^7^. Nevertheless, the impact and burden of lung cancer attributed to smoking prevalence on global healthcare systems is likely to persist for decades^8^.

The causal link between smoking behaviour and risk of lung cancer is well established^9–12^. However, ecological models that have incorporated smoking data have focused on mortality (rather than incidence), and being projection models, are optimised for predictive accuracy as opposed to estimation of the exposure–lag response^13^. The latter calls for *explanatory* modelling; this will be invaluable to policy makers for estimating the effect of changing the proportion of smokers in a population, thus facilitating strategic and robust planning^14^.

Here we present an ecological modelling study with the overarching aim of estimating the exposure–lag response of smoking prevalence on lung cancer incidence, while controlling for confounding variation attributable to country, age group, gender and population at risk. We chose smoking prevalence as our index of “exposure”, since this is the predominant diver of lung cancer incidence in a population^15^ Recent lung cancer incidence and population at risk estimates are matched by country, age group and gender to a complete 30-year exposure history of smoking prevalence data. We stipulated the following research questions:

1. How does the exposure–response of smoking prevalence on lung cancer incidence vary by lag period?
2. How does the lag–response of smoking prevalence on lung cancer incidence vary by smoking prevalence?
3. How does the cumulative lag response of smoking prevalence on lung cancer incidence vary by smoking prevalence?

## Material and methods

**Figure 1:**
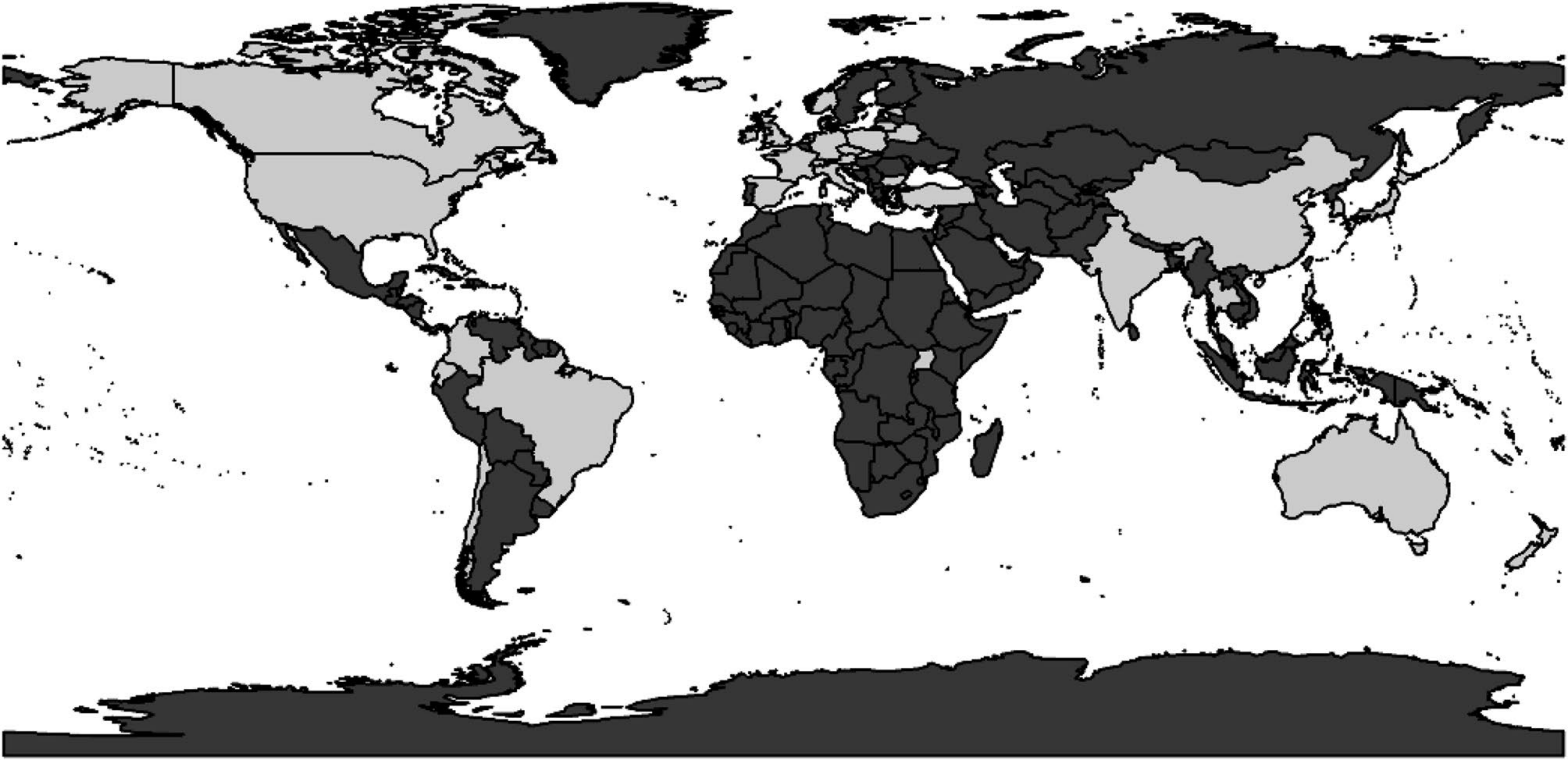
Countries included in our analysis (shaded grey). The full list of countries include: Australia, Austria, Bahrain-Bahraini, Belarus, Brazil, Bulgaria, Canada, Chile, China, Colombia, Costa Rica, Croatia, Cyprus, Czech Republic, Denmark, Ecuador, Estonia, France, Germany, Iceland, India, Ireland, Israel, Italy, Japan, Korea, Kuwait, Lithuania, Malta, Netherlands, New Zealand, Norway, Philippines, Poland, Slovakia, Slovenia, Spain, Switzerland, Thailand, Turkey, Uganda, UK and the United States of America. Generated using R package “maptools” version ‘1.0.1’ (R version 4.0.2).

Annual age group (40–44, 45–49, 50–54, 55–59, 60–64, 65–69, 70–74, 75–79 years) and gender (M/F) specific lung cancer incidence and corresponding populations at risk were obtained from the Cancer Incidence in Five Continents plus (CI5 plus) database for the period 2010 through 2012 inclusive from 105 cancer registries across 43 countries^16^ (Fig. 1). We excluded data for 19 cancer registries which were ethnicity grouped subsets of larger registries (for example, we retained USA-SEER, but discarded USA-SEER-White and USA-SEER-Black).

We obtained smoking prevalence estimates from the Institute for Health Metric and Evaluation (IHME) for the period 1980 through 2012 inclusive, matched by country, gender and age group^17,18^. The latter was matched retrospectively to properly characterise the smoking prevalence exposure history. For example, lung cancer incidence in the 75–79 age group in 2012 was matched with smoking prevalence for the 75–79 age group in 2012, the 70–74 age group in 2008 etc.

In order to model the temporal dependency between changes in smoking prevalence in a population and lung cancer incidence, we employed a distributed lag non-linear model (DLNM)^19,20^. Such models allow non-linearity in the bi-dimensional exposure–lag response surface and therefore offer a significant advantage to traditional approaches^21,22^. We define the statistical unit of analysis on demographic strata, *i*, (group of individuals, from country, C, in a given age group, A, of a given gender, G) at time *t* (outcome year = 2010, 2011, 2012).

Our statistical model took the form:

1 $$ \begin{aligned} & Y_{it} \sim Negative \;binomial \left( {\mu_{it} } \right), \\ & log\left( {\mu_{it} } \right) = \alpha + log\left( {P_{it} } \right) + {\varvec{\beta}}S_{it,l} + {\varvec{\gamma}}C_{it} + {\varvec{\delta}}A_{it} + \eta G_{it} + {\varvec{\lambda}}O_{it} + {\varvec{\nu}}A_{it} G_{it} \\ \end{aligned} $$

where $$Y_{it}$$ is the observed lung cancer incidence, $$\mu_{it}$$ the expected (mean) lung cancer incidence, $$\alpha$$ the model intercept, and $$P_{it}$$ the population at risk. $$S_{it,l}$$ is a cross-basis matrix for smoking prevalence, with *l* representing the lag (= 0,1,2…30 years) and $${\varvec{\beta}}$$ a vector of coefficients. $$C_{it} , A_{it}$$ and $$O_{it} $$are fixed effect categorical variables for country, age group, and outcome year respectively, with $${\varvec{\gamma}},{\varvec{\delta}}$$ and $${\varvec{\lambda}}$$ each representing the respective vectors of coefficients. $$A_{it} G_{it}$$ represents an interaction term between age group and gender with $${\varvec{\nu}}$$ its vector of coefficients. *G*_*it*_ is a binary variable representing gender, set to 1 for male and 0 otherwise, with $$\eta$$ its corresponding coefficient. The cross-basis matrix $$S_{it,l}$$ was constructed using restricted cubic splines (i.e. natural splines) with 4 pre-specified degrees of freedom for both the lag and exposure bases. Spline functions were used as opposed to simple polynomial terms since the latter will not fit many functional forms^23^. Restricted cubic splines were chosen over cubic splines for two reasons: (1) the function is constrained to be linear in the tails (before the first knot and after the last knot) improving performance; and (2) only *k* − 1 parameters must be estimated (besides the intercept) as opposed to *k* + 3 parameters with the unrestricted cubic spline^23^.

The most recent year of outcome data (i.e. lung cancer incidence) in CI5 plus is 2012. The decision to include earlier outcome years must be weighed against the reduction in maximal lag period (this must remain constant when modelling several outcome years). We opted for a 30-year lag period^15,24^ which enabled us to include data from three lung cancer outcome years, namely 2010, 2011 and 2012. Accordingly, lung cancer outcomes in 2010, 2011 and 2012 utilised lagged smoking prevalence histories of 1980–2010, 1981–2011 and 1982–2012 respectively. We applied a constraint in the model described in Eq. (1) by excluding the intercept in the lag dimension of the cross-basis term. This had the effect of fixing the Incidence Rate Ratio (IRR, see below) to 1 at lag = 0 years, implying that changing the proportion of smokers in a population has no immediate effect on lung cancer incidence within the same year. The natural logarithm of *P*_*it*_ constitutes the model offset to account for varying populations at risk. Estimates of parameters was performed using full maximum likelihood. We pre-specified three additional competing models which were reduced forms of Eq. (1), those being: (1) omission of the interaction term between gender and age; (2) reduction from 4 to 3 degrees of freedom for each of the restricted cubic splines in the cross-basis term; and (3) both of the above. However, the full model (i.e. Eq. 1) outperformed these competing models as verified by Akaike’s Information Criterion (AIC). To check the assumptions of our selected model, we computed scaled (quantile) residuals using a simulation-based approach^25^.

The estimated coefficients and variance–covariance matrix of ${S}_{it,l}$ were used to predict the exposure–lag response. We used the sandwich estimator to correct the variance–covariance matrix of ${S}_{it,l}$ using registry as a cluster variable^26–28^. Accordingly, standard errors were robust to autocorrelation and heteroskedasticity. Effects are presented as the incidence rate ratio (IRR) (± 95% confidence intervals) to quantify the direction and magnitude of the exposure–lag–response. The IRR represents the ratio of lung cancer incidence rate in the “exposed” group (i.e. specified increment in smoking prevalence compared to some reference level) to the lung cancer incidence rate in the ‘non-exposed” group (i.e. smoking prevalence at the reference level). Accordingly, the IRR represents the effect that changing the proportion of smokers in a population has on lung cancer incidence. Because we accounted for other covariates, the predicted changes in lung cancer incidence are “standardized” and represent the average effect, pooled over age groups, gender, country and outcome year. For analysis and interpretation, we set the reference level to 50% smoking prevalence, though corresponding figures with the reference level set at the maximum smoking prevalence value from the exposure history are provided in the supplementary material. We also computed cumulative incidence rate ratios (IRR_cum_) at each lag, by summing the logarithm of IRR’s from previous lags. These represent the incremental effects of IRR’s for a given smoking prevalence history. Since our study design incorporated multiple exposure events (i.e. smoking prevalence histories) IRR and IRR_cum_ for the exposure–lag response are estimated from the “backward-perspective”^19,20^.

All statistical analyses was performed in R version 4.0.2^29^ relying heavily on the packages dlnm, MASS and DHARMa^19,25,30^. Fully reproducible R code is included in the supplementary material.

## Results

The number of lung cancer cases and corresponding populations at risk, aggregated across registries and countries, is shown in Table 1. Model checking plots of the simulated residuals indicated a good fit to the observed data with residuals close to the 1:1 line of observed vs. expected (supplementary information Fig 1).

**Table 1:** Number of lung cancer cases and corresponding populations at risk by year, gender and age group.

Years	Gender	Age group (years)	Number of cases	Population at risk
2010	Female	40–44	1844	22,856,931
2010	Female	45–49	5245	23,960,807
2010	Female	50–54	10,221	23,072,194
2010	Female	55–59	14,246	20,691,616
2010	Female	60–64	20,680	18,415,911
2010	Female	65–69	24,933	14,027,181
2010	Female	70–74	25,709	11,412,962
2010	Female	75–79	25,272	9,774,883
2010	Male	40–44	1874	22,599,303
2010	Male	45–49	5879	23,513,387
2010	Male	50–54	13,273	22,401,557
2010	Male	55–59	21,862	19,638,456
2010	Male	60–64	31,335	17,208,726
2010	Male	65–69	37,182	12,590,896
2010	Male	70–74	38,012	9,572,416
2010	Male	75–79	35,099	7,372,029
2011	Female	40–44	1779	22,515,379
2011	Female	45–49	4888	23,205,727
2011	Female	50–54	10,140	22,878,792
2011	Female	55–59	14,662	20,711,581
2011	Female	60–64	20,963	18,732,935
2011	Female	65–69	25,533	14,106,947
2011	Female	70–74	25,973	11,342,181
2011	Female	75–79	24,426	9,590,696
2011	Male	40–44	1857	22,203,201
2011	Male	45–49	5298	22,795,845
2011	Male	50–54	12,433	22,235,507
2011	Male	55–59	21,238	19,662,798
2011	Male	60–64	30,814	17,480,755
2011	Male	65–69	36,380	12,728,774
2011	Male	70–74	37,431	9,580,269
2011	Male	75–79	33,621	7,314,162
2012	Female	40–44	1705	22,288,992
2012	Female	45–49	4665	22,633,652
2012	Female	50–54	10,155	22,775,001
2012	Female	55–59	15,496	20,969,853
2012	Female	60–64	20,874	18,565,286
2012	Female	65–69	27,149	14,989,704
2012	Female	70–74	26,907	11,532,883
2012	Female	75–79	24,943	9,558,073
2012	Male	40–44	1741	21,950,722
2012	Male	45–49	4822	22,243,431
2012	Male	50–54	11,989	22,149,777
2012	Male	55–59	21,126	19,897,569
2012	Male	60–64	30,103	17,277,257
2012	Male	65–69	37,420	13,561,101
2012	Male	70–74	37,526	9,787,986
2012	Male	75–79	33,359	7,344,897

Cases and population at risk counts are aggregated across registries and countries.

**Figure 2:**
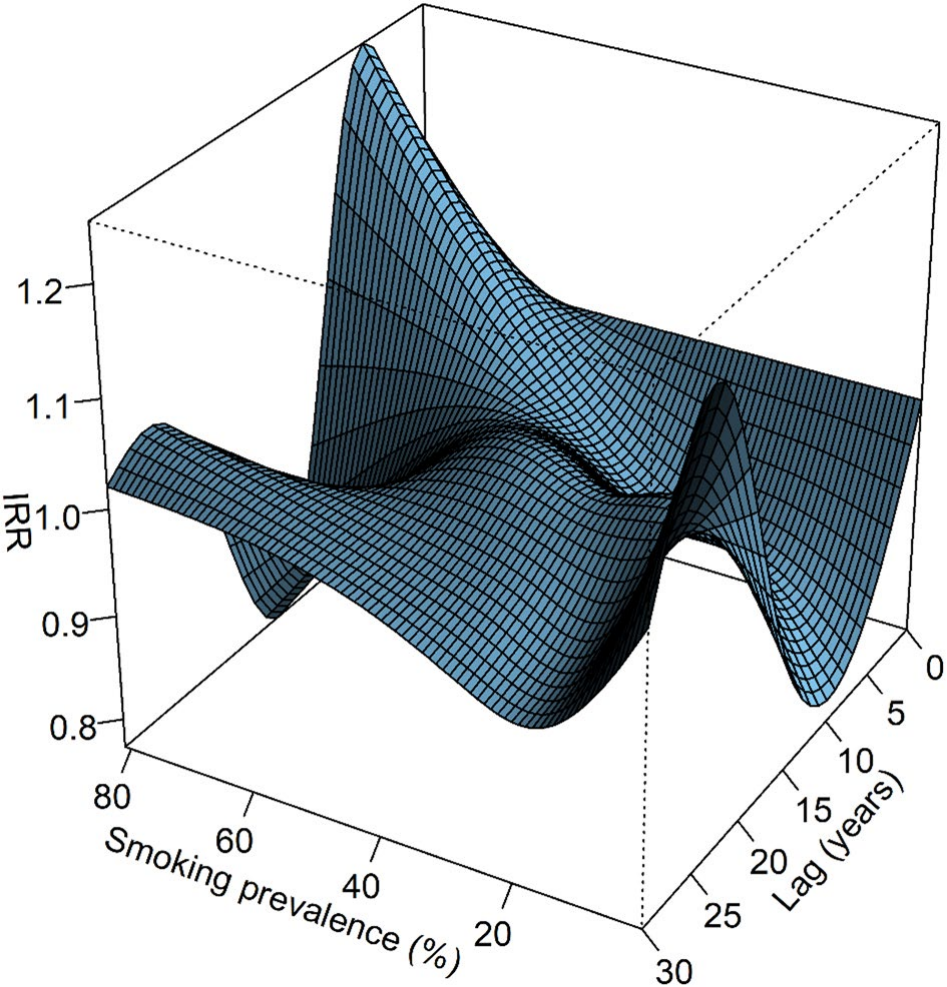
Bi-dimensional exposure–lag response surface showing joint effect of lag (years) and smoking prevalence (%) on predicted incidence rate ratio (IRR) of lung cancer. Effects are relative to the reference level of 50% smoking prevalence.

Across the bidimensional surface of all smoking prevalence and lag values, minimum IRR was 0.77 [95% CI 0.74, 0.83] occurring at smoking prevalence = 81% and lag = 16 years, whilst maximum IRR was 1.25 [95% CI 1.17, 1.34] occurring at smoking prevalence = 81% and lag = 6 years (Fig. 2). There was a clear interaction effect between increments in smoking prevalence and lag on the IRR, whereby the response to changes in smoking prevalence were dependant on the lag and vice versa.

**Figure 3:**
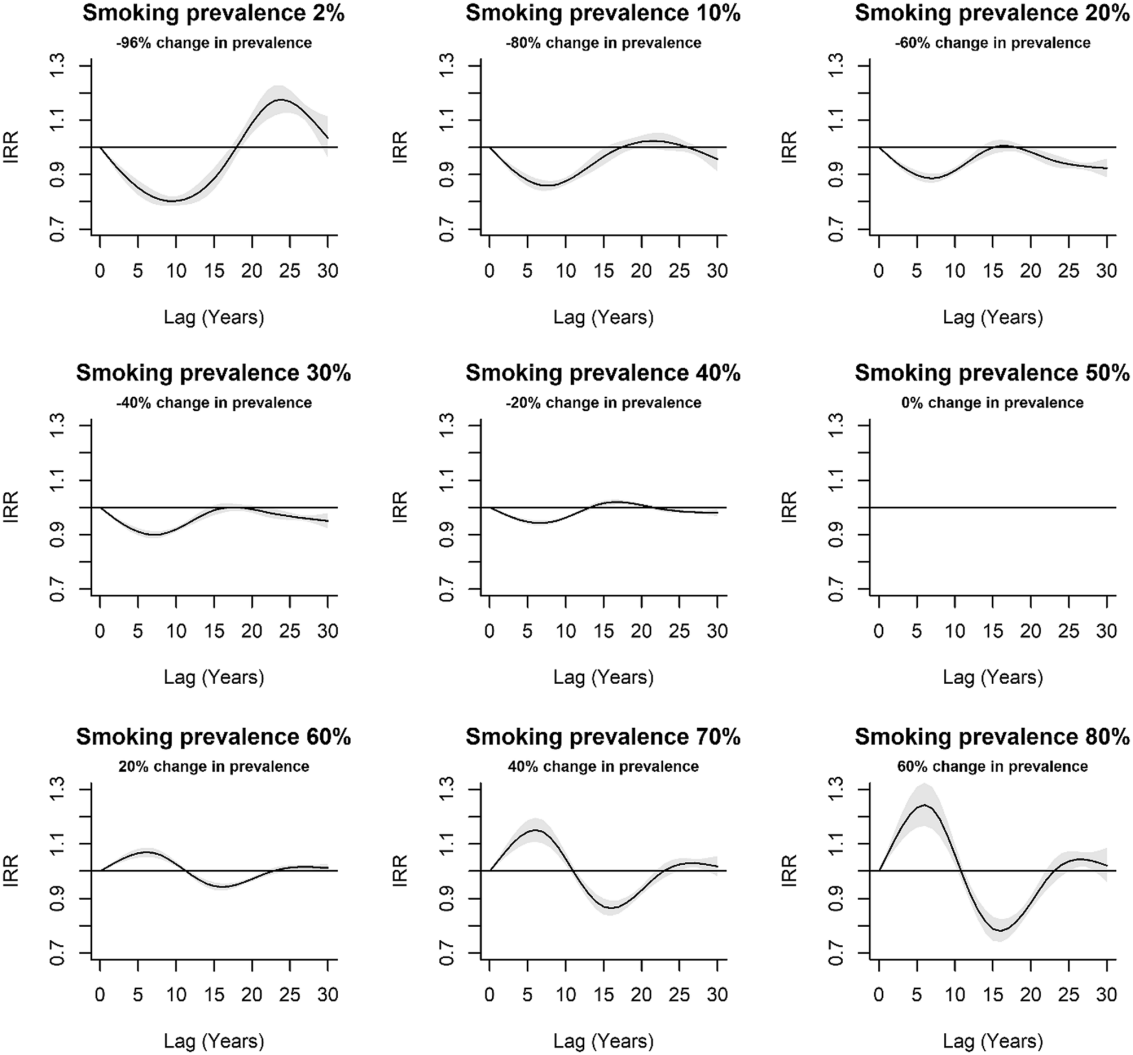
Estimated lag response of the incidence rate ratio (IRR) and 95% confidence intervals for specified increments in smoking prevalence (%). IRR > 1 indicates a positive association whilst IRR < 1 indicates a negative association. Increments in smoking prevalence are relative to the reference level of 50% smoking prevalence. For example, for the panel entitled smoking prevalence 60%, this implies a percentage increase of 20% smoking prevalence in the population ((60–50)/50 × 100).

The lag response was wave-like, with the direction and magnitude varying according to the direction and magnitude of the increment in smoking prevalence compared to the reference level (i.e. 50% smoking prevalence) (Fig. 3). Positive increments in smoking prevalence produced a lag–response exhibiting an inverted U-shape between approximately 0 and 11 years lag, U-shaped between approximately 11 and 23 years lag, and largely flat between approximately 23 and 30 years lag. Lag–response curves for negative increments in smoking prevalence were approximate inversions of the lag response for positive increments in smoking prevalence. Accordingly, the lag response was U-shaped at recent lags, followed by an inverted U-shape at later lags.

**Figure 4:**
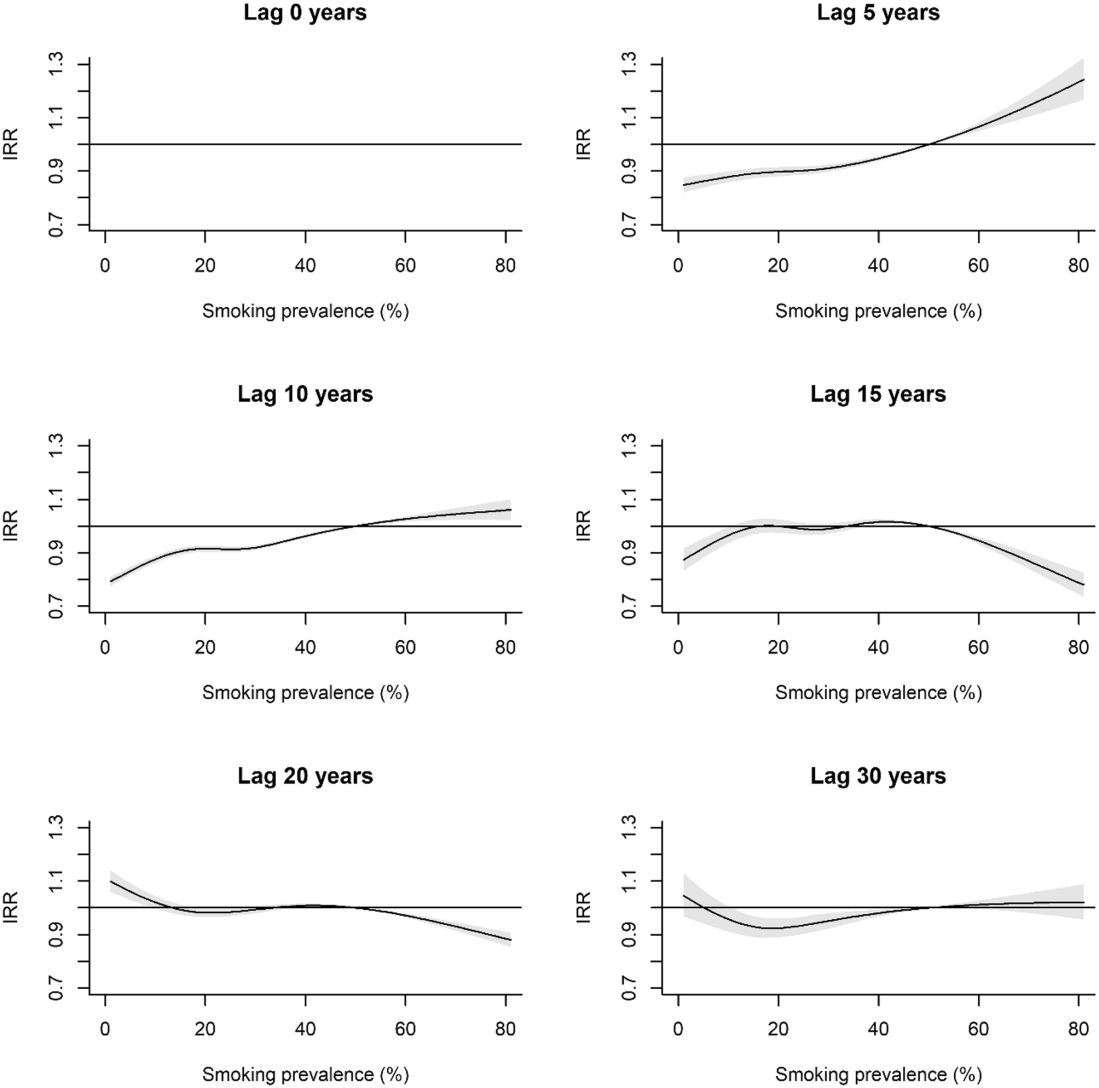
Estimated smoking exposure response of the incidence rate ratio (IRR) and 95% confidence intervals for specified lag periods (years). IRR is computed using the reference level of 50% smoking prevalence (i.e. the mean across all lags and countries). Accordingly, the IRR = 1 at smoking = 50% for all panels on the plot.

The exposure–response curves varied according to the lag period (Fig. 4). For example, at lag = 5 years, the curve form showed an approximately exponential increase, at lag = 10 years approximately linear increase, and lag = 15 years approximately parabolic. At recent lags e.g. 5 and 10 years, positive and negative increments in smoking prevalence compared to the reference level clearly show an increased (IRR > 1) and decreased (IRR < 1) incidence of lung cancer respectively. Interestingly, the model predicts IRR < 1 for positive increments in smoking exposure at lags of 15 and 20 years.

**Figure 5:**
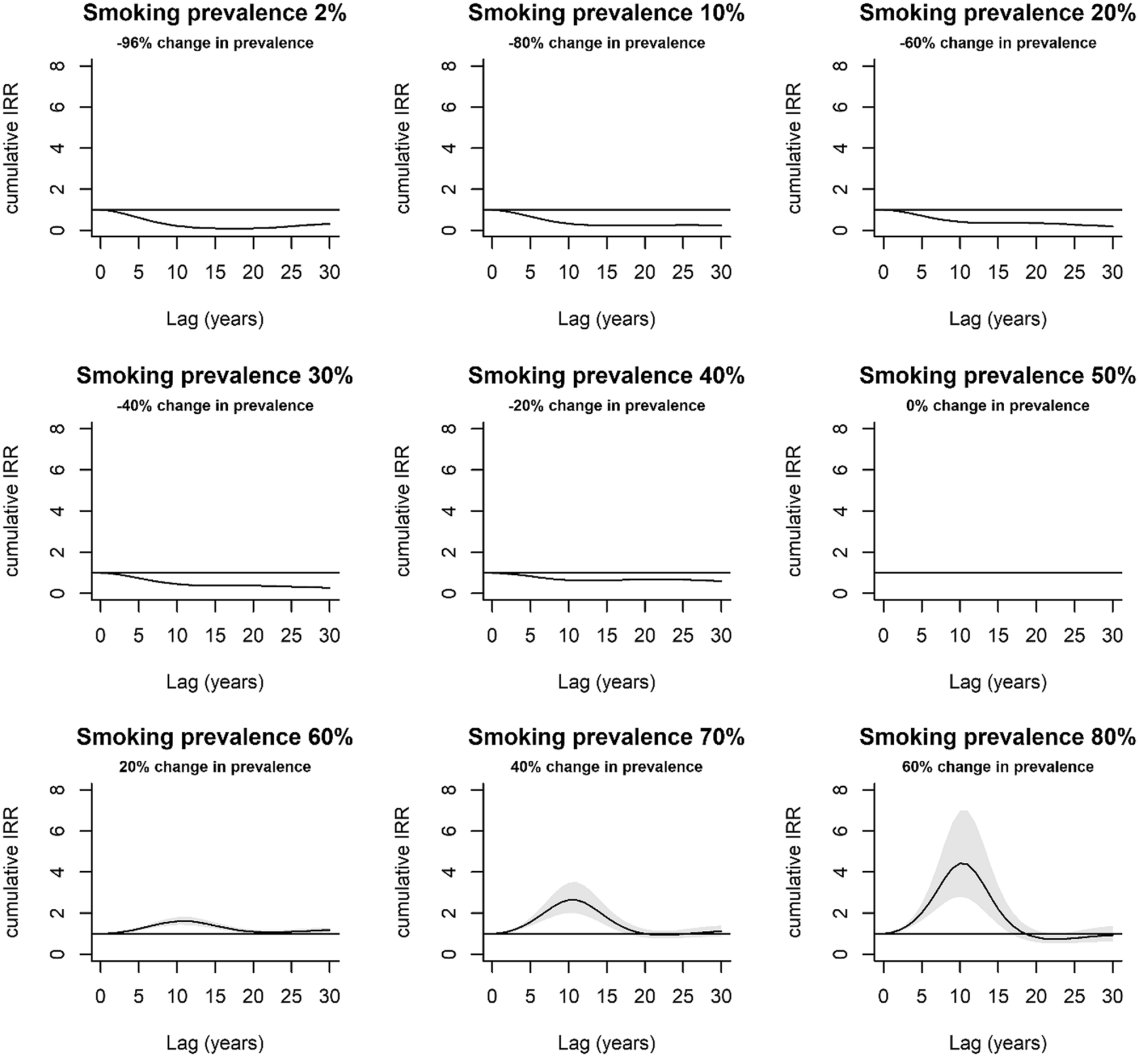
Estimated lag response of the cumulative incidence rate ratio (IRR_cum_) for specified increases in smoking prevalence (%). Increments in smoking prevalence are relative to the reference level of 50% smoking prevalence. For example, for the panel entitled smoking prevalence 60%, this implies a percentage increase of 20% smoking prevalence in the population ((60–50)/50 × 100)).

Minimum IRR_cum_ was 0.07 [95% CI 0.05, 0.09] at smoking prevalence = 1% and lag = 17 years, whilst maximum IRR_cum_ was 4.67 [95% CI 2.90, 7.50] at smoking prevalence = 81% and lag = 10 years. Increments above and below the reference level of 50% smoking prevalence was associated with an increased and decreased IRR_cum_ respectively, with the magnitude of the effect varying across the lag period (Fig. 5). Accordingly (with the exception of high smoking prevalence), across the entire lag period, interval estimates for IRR_cum_ were ≥ 1 and ≤ 1 for positive and negative increments in smoking prevalence respectively. The cumulative lag response was approximately bell-shaped for increments in smoking prevalence above the reference level, with the peak IRR_cum_ occurring at approximately lag = 10 years. The magnitude of the effect increased with greater increases in smoking prevalence relative to the reference level.

## Discussion

In this contribution, we investigated the effect that changing the proportion of smokers in a population (i.e. smoking prevalence) has on standardized lung cancer incidence. This was achieved using a DLNM to quantify non-linear exposure–response dependencies and delayed effects^20^. We utilised data from 105 cancer registries across 43 countries and a complete smoking prevalence exposure history of 30 years. By incorporating data from a large number of countries over an extended time period, we were able to capture a wide range of smoking prevalence values, enabling us to fully capture the exposure–lag response.

The associations between lung cancer incidence and increments in smoking prevalence reflected in our effect plots adds to the current body of evidence that smoking prevalence is a major driver of lung cancer incidence in a population^31–34^. Heloma et al.^35^ showed an approximately positive linear association between smoking prevalence at 20 years lag and current lung cancer incidence in a male Finnish population as compared to the clearly non-linear response reported herein (Fig. 4; lag = 20 years). Our results also imply that for a given change in smoking prevalence in a population, the lag period might be shorter than previously thought (e.g. 20 years^8^; 30 years^15^), though we emphasize that this is dependent on the initial smoking prevalence rate as well as the magnitude and direction of any changes.

For positive increments in smoking prevalence, one might expect the IRR in the lag response curves to increase above 1 (implying a positive association) to some peak, before exponentially declining to plateau at IRR = 1 towards the end of the lag period. It follows that the reverse would be expected for negative increments in smoking prevalence. Our results in part show this response, though the IRR < 1 (for positive increments) and IRR > 1 (for negative increments) in the later lags is somewhat unexpected. A potential explanation for this paradox is offered by the harvesting hypothesis. Depletion of the pool of susceptible individuals after a period of exposure renders the observed population healthier than a counterfactual population^36^. Such harvesting should not be interpreted as a true protective association at longer lags, but rather an artefact due to a change in the underlying population^36^ The decline in IRR below 1 at lag 15 and 20 years if smoking prevalence is increased above the reference level of 50% might then represent the harvesting effect described above, viewed from a different perspective.

The exposure–response and lag–response trends are reflected in the cumulative effects plots (Fig. 5). The increase in magnitude of the response with larger increments in smoking prevalence is consistent with the positive relationship between smoking prevalence and lung cancer incidence. For positive increments in smoking prevalence, the decline in IRR_cum_ after the peak at approximately 10 years might be a direct consequence of the harvesting effect detailed above. For positive increments in smoking prevalence, the IRR_cum_ interval estimates never fall below 1 (except at very high increments in smoking prevalence) for the entire lag period implying the high risk (IRR > 1) at earlier lags has compensated for any apparent protective association (IRR < 1) at later lags. Conversely, for negative increments in smoking prevalence, the interval estimate for IRR_cum_ is less than 1 for the entire lag period, implying any apparent positive association at later lags (IRR > 1) has been compensated by negative associations (IRR < 1) at earlier lags.

Although our model included covariates (age group, gender, country, outcome year) to control for confounding variation, further analyses might consider additional known predictors of lung cancer such as ethnicity^37^ or socioeconomic status^38^. Furthermore, our model assumes that the exposure–lag response of smoking prevalence on lung cancer incidence is independent of our modelled covariates. This is a strong assumption; for example Chang et al.^11^ reported a significant age-by-pack-years interaction, whilst studies in Asia have reported a higher female to male ratio of the relative risk compared to non-Asian studies^15,39,40^. One way of relaxing such assumptions might be to adopt a two-stage design^41^. In the first stage, a series of covariate-specific DLNM’s are fitted, after which the cross-basis terms $S_{it,l}$ are reduced to simpler sets of one-dimensional coefficients and covariances for the exposure- and lag–dimensions respectively. In the second stage, these are then pooled using meta-analysis.

We chose smoking prevalence as our smoking index since this is a strong predictor of lung cancer incidence in a population^15^. Although it is possible to include multiple cross-basis terms in DLNM’s, we decided against this to avoid the issue of multicollinearity, since our principle aim was to isolate the exposure–lag response of smoking prevalence on lung cancer incidence. Nevertheless, future studies might consider other indices of smoking history such as cigarette sales. Recent work has extended the DLNM framework through the use of penalized splines within generalized additive models (GAM)^42^, which provide built‐in model selection procedures and the possibility of accommodating assumptions on the shape of the lag structure through specific penalties^43^. It has been shown that this penalized extension to DLNM’s provides greater flexibility and improved inferential properties^43^ and so this approach might be considered in future works.

Using our model, the analyst can estimate IRR_cum_ for given smoking prevalence scenarios (i.e. changes in the proportion of smokers in a population) over desired future time periods. IRR_cum_ point estimates and confidence intervals are easily obtained from our model predictions (R code in supplementary material), by specifying a smoking prevalence reference level, the increment relative to the reference level, and the future time period. For example, reducing the percentage of smokers in a population from 50 to 40% (i.e. 20% reduction) for a period of 10 years hence is estimated to produce a 54% [95% CI 45%, 64%] reduction in the lung cancer incidence rate (IRR_cum_ = 0.65 [95% CI 0.61, 0.69]). This can be easily demonstrated by use of interactive plotting software. We have used the R Shiny package^44^ to demonstrate the utility of our model for users to examine the effect that can be expected if the smoking prevalence is changed over a future time span of 30 years (https://abehzadnia.shinyapps.io/LungCa_Exposure_lag_response-Smith_et_al_2021/). One can also accommodate more complex scenarios where the smoking prevalence rate changes across time. The fact that the analyst need only input smoking prevalence to make these predictions makes our model particularly attractive since projections are widely available for many countries^3^.

It should be reminded that the smoking prevalence data we used herein are modelled estimates, and therefore subject to inherent uncertainties, discussed well elsewhere^1^. Our DLNM requires complete ordered data over the time series, and to our knowledge, no such global dataset of *observed* data exists. However, the smoking dataset used in this analysis is from a highly credible source which has undergone a rigorous validation process and was hence chosen because we believed it to be the most suitable for our study.

A final word of caution is that interpretation of time-varying IRR’s is certainly non-trivial and may not convey a sense of the true burden associated with changing smoking prevalence in populations. For example, if the baseline lung cancer incidence rate is very small, even a relatively large IRR might not lead to a big difference in cumulative lung cancer incidence between exposed and unexposed groups. Accordingly, computation of absolute risks in future work would be particularly useful for decision makers.

## Conclusions

This was the first study to quantify the effect that changing smoking prevalence in a population has on lung cancer incidence. The exposure–response varied by lag period, whilst the lag–response varied according to the magnitude and direction of changes in smoking prevalence in the population. For the cumulative lag–response, increments above and below the reference smoking prevalence level was associated with an increased and decreased IRR_cum_ respectively, with the magnitude of the effect varying across the lag period. By isolating the exposure–lag response, our model can be used to perform simple “what-if” analyses; that is, assessing changes in lung cancer incidence as a result of modifying the proportion of smokers in a population. We hope our work constitutes a preliminary step towards providing policy makers with meaningful indicators to inform national screening programme developments. To that end, we have implemented our model as an easy-to-use shiny app and provided an example of its use.

## Supplementary Information

Supplementary Figures.

Supplementary Data.

Supplementary Code.
