# Supplementary material for: Exposure–lag response of smoking prevalence on lung cancer incidence using a distributed lag non-linear model
Source: Sci Rep. 2021 Jul 14;11:14478. doi: 10.1038/s41598-021-91644-y (PMC8280159; doi:10.1038/s41598-021-91644-y)
Supplement: Supplementary file 1 — Supplementary Figures. [file 41598_2021_91644_MOESM1_ESM.pdf]

Exposure-lag response of smoking prevalence on lung cancer incidence using a distributed lag non-linear model

Daniel Robert Smith[1]*, Alireza Behzadnia[2], Rabbiaatul Addawiyah Imawana[1], Muzammil Nahaboo Solim[3], Michaela Louise Goodson[1]

Contact details:

Daniel Robert Smith *Corresponding Author
ORCID id. 0000 0002 3916 8041
Email: daniel.smith6@newcastle.edu.my

Alireza Behzadnia
Email: alireza.behzadnia@doctors.org.uk

Rabbiaatul Addawiyah Imawana
Email: r.a.binti-imawana2@newcastle.ac.uk

Muzammil Nahaboo Solim
Email: m.nahaboosolim@nhs.net

Michaela Louise Goodson
Email: michaela.goodson@newcastle.edu.my

Institutions:

[1]Newcastle University Medicine Malaysia,
No. 1, Jalan Sarjana 1,
Kota Ilmu, EduCity@Iskandar,
79200 Iskandar Puteri,
Johor,
Malaysia.

[2]Histopathology department,
Leeds Teaching Hospital
NHS Trust,
Beckett Street,
Leeds,
West Yorkshire,
LS9 7TF,
UK

[3]The James Cook University Hospital,
Marton Road,
Middlesbrough,
TS4 3BW,
UK.

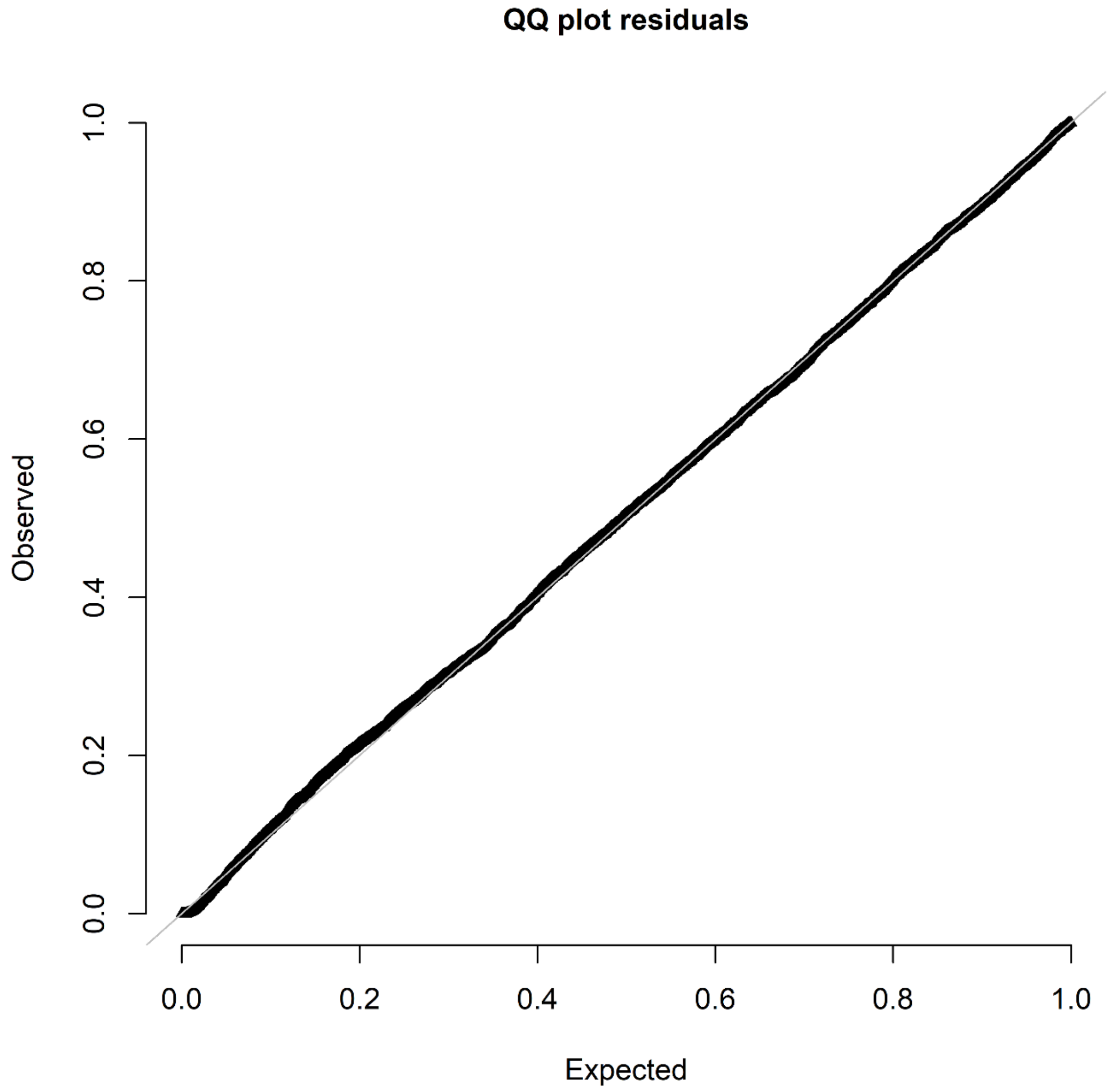

Supplementary information fig 1. Quantile-Quantile plot of scaled residuals from our fitted model computed using a simulation-based approach. [24]

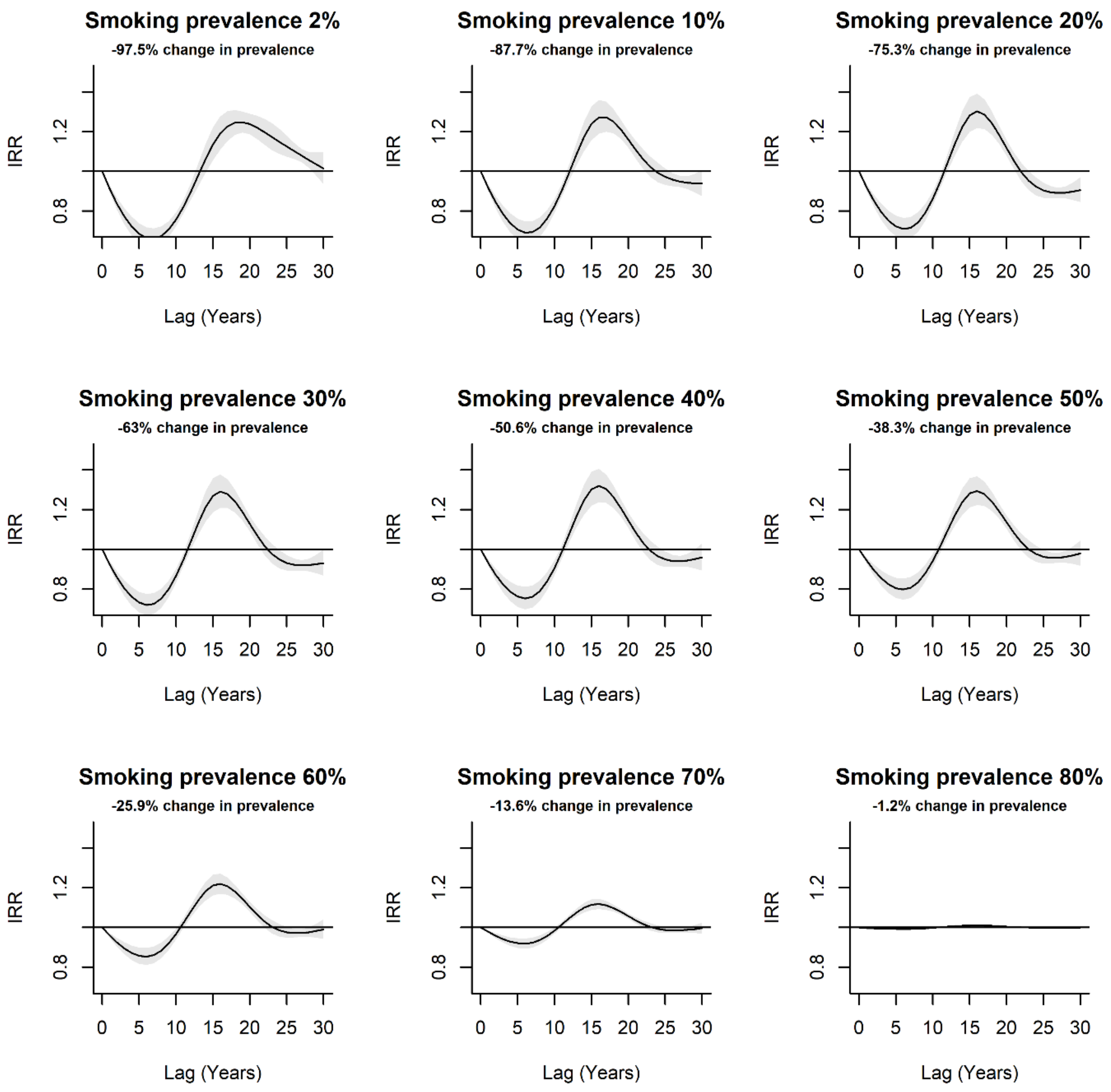

SI fig 2. Estimated lag response of the incidence rate ratio (IRR) and 95 % confidence intervals for specified increments in smoking prevalence (%). IRR>1 indicates a positive association whilst IRR<1 indicates a negative association. Increments in smoking prevalence are relative to the reference level of 81 % smoking prevalence.

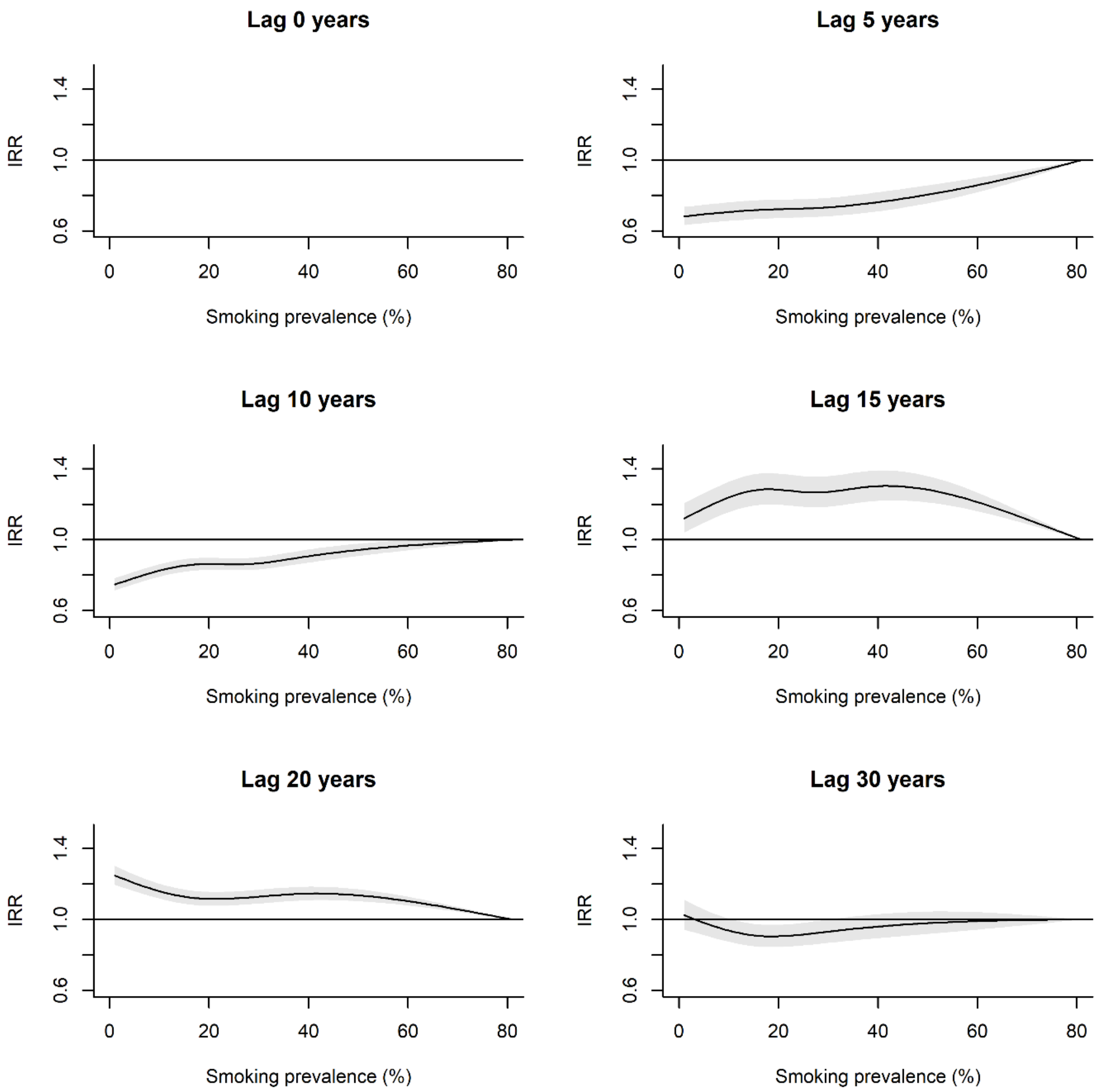

SI fig 3. Estimated smoking exposure response of the incidence rate ratio (IRR) and 95% confidence intervals for specified lag periods (years). IRR is computed at the reference level of 81 % smoking prevalence.

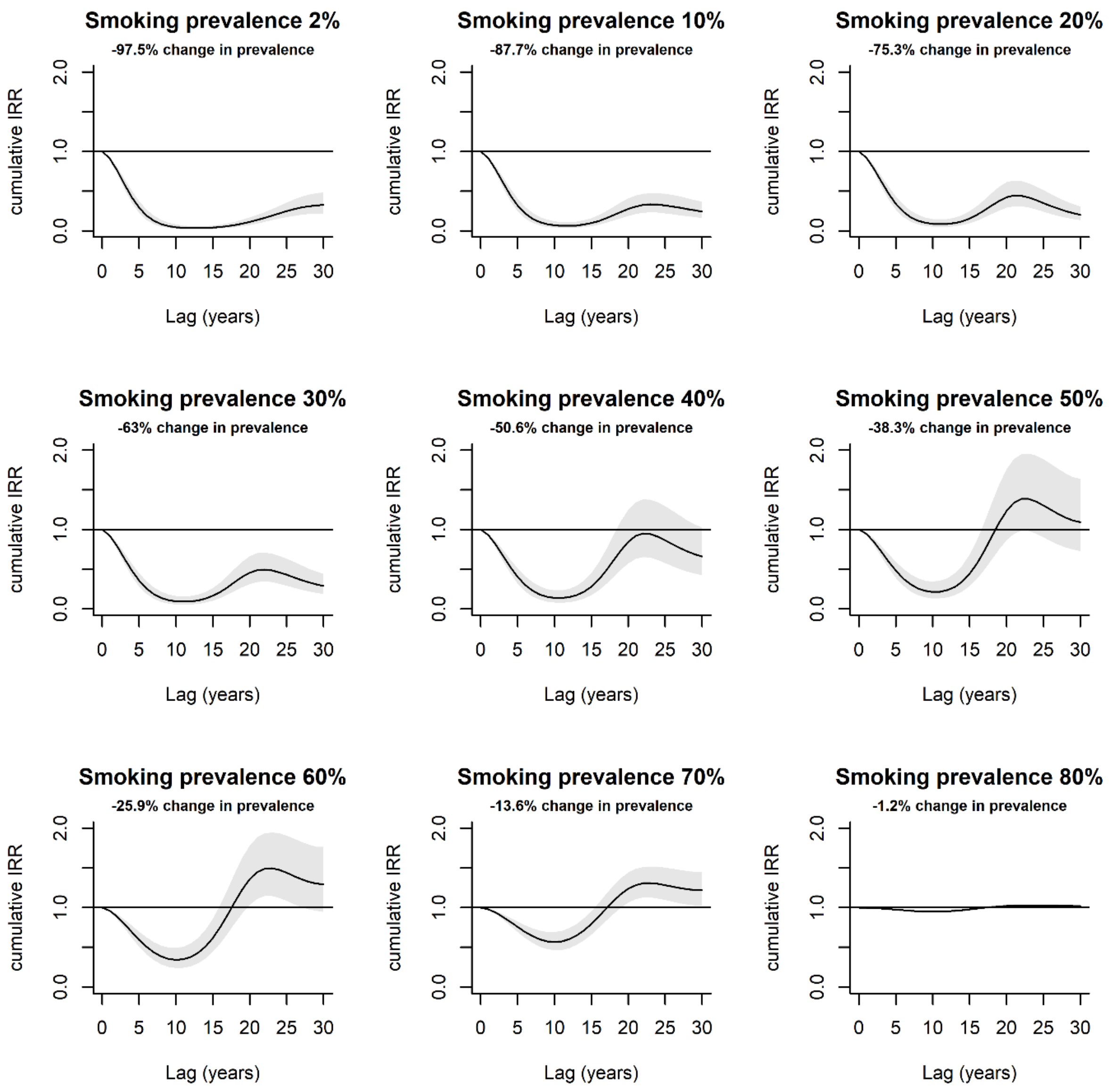

SI fig 4 Estimated lag response of the cumulative incidence rate ratio (IRR_{cum}) for specified increments in smoking prevalence (%). Increments in smoking prevalence are relative to the reference level of 81 % smoking prevalence.
